# Poor Diet in Shift Workers: A New Occupational Health Hazard?

**DOI:** 10.1371/journal.pmed.1001152

**Published:** 2011-12-27

**Authors:** The PLoS Medicine Editors

## Abstract

The *PLoS Medicine* Editors discuss the link between shift work, diet, and type 2 diabetes, and argue that unhealthy eating should be considered a new form of occupational hazard.

As a specialty, occupational health has changed dramatically from its inception when industrial hazards predominated. It retains, however, a critical role in safeguarding workers and their health rights. As we argued in an editorial in 2007 [1], “the right to work in safety is a basic human right, and protection of the health of workers is ultimately of benefit to all of society”. A paper published earlier this month in *PLoS Medicine* by An Pan and colleagues provides a new angle on the link between wider public health and occupational health, specifically the effect of shift work.

According to the International Agency for Research on Cancer (part of the World Health Organization) [2], about 15%–20% of the working population in Europe and the US is engaged in shift work (defined by the International Labor Organisation as “a method of organization of working time in which workers succeed one another at the workplace so that the establishment can operate longer than the hours of work of individual workers” [3]. Shift work occurs in virtually all industries—but one in which it is unavoidable, given the need for 24-hour coverage, and where virtually every worker will be exposed at some point in their working life, is the health-care industry. An Pan and colleagues' paper [4] provides compelling evidence of the link between shift work and type 2 diabetes in women, with the effect partly, but not exclusively, moderated by BMI. The paper is one of many that have come out of the long-running US Nurses Health Study (NHS), which started in 1976 and expanded in 1989 (and is now recruiting for its next phase, NHS3) [5]. The association between shift work and diabetes has been suggested before [6]–[8], but this study, with 18–20 years of follow up, is the best evidence yet for this link, with an increasing risk of type 2 diabetes in the first and second cohorts of the NHS as duration of shift work increased. As the authors note, there is now good evidence that “[p]roper screening and intervention strategies in rotating night shift workers are needed for prevention of diabetes.” The authors speculate on the mechanisms that might underlie this association, which include disruption of the circadian rhythms that regulate metabolic and cardiovascular systems, a negative effect on diet and exercise, and an effect on both quality and quantity of sleep.

Suggesting as it does that working patterns are a specific risk factor for obesity and type 2 diabetes—currently at epidemic proportions in the developed world and likely to become so soon in the less-developed world—the authors draw attention to some intriguing possibilities for where the field of occupational health might now fruitfully focus its efforts. Although some of the effects of shift work are probably unavoidable (such as disruption of circadian rhythms, although even this effect can be ameliorated somewhat by careful management of shifts), others, such as eating patterns, are obvious targets for intervention. It would, however, require a change in thinking and an acceptance that occupational health needs to move into territory more personal than before: the diet of workers.

But such a change would not be unthinkable. There is a wealth of evidence that shift workers struggle to eat healthily and occupational websites and blogs abound with “tips” on eating better. Even for day workers, employers are encouraged to help their workers eat healthily. However, seriousness of these efforts varies. For example, Canadian Occupational Health [9] (which is run with the involvement of employers, government, and labor) is typical in its advice on the development of a healthy eating program: “As always, these programs should be part of a complete workplace health program and should not take resources or attention away from workplace hazards that may be present.” It's interesting to contrast this advice with that on, for example, reducing exposure to tobacco smoke, which is generally much more prescriptive; indeed it is interesting to note how the wording makes the assumption that eating unhealthily is *not* itself a “hazard.”

As the world of work becomes increasingly 24 hour, shift work will become more common. And if the data from this and other studies are to be taken at face value, shift work has the potential to accelerate the progression of the global epidemic of obesity and diabetes. Obviously, diet is only one component in the pathway to diabetes, but, unlike the metabolic consequences of a deranged circadian rhythm it is potentially amenable to easy intervention.

However, healthy eating has proved to be a surprisingly difficult nut to crack with simple persuasion alone; consumers worldwide are often unwilling to change their habits voluntarily, even when they are familiar with the risks of unhealthy eating. The UK government, for example, has recently come in for much criticism [10] for their policy which, by simply repeating what consumers already know about the need for a healthy diet, seems to be more tailored to the needs of the food industry than to the health of the population as a whole. Increasingly, public health advocates feel that concrete action is needed: governments need to legislate to improve the habits of consumers and take specific steps to ensure that it is easier and cheaper to eat healthily than not. Could workplaces, specifically those who employ shift workers, lead the way (or be required to lead the way) in such a drive? One workplace, the Cleveland Clinic, has done just that (as well as introducing incentives for exercise and smoking cessation) and seen specific increases in health of employees [11]. From the accumulating evidence, it seems that unhealthy eating could legitimately be considered a new form of occupational hazard. Such a perspective is not so far away from the thinking that led to the first laws that regulated worker safety, and arguably the effect on public health may be even greater.
